# Preeclampsia and intrauterine growth restriction: Role of human umbilical cord mesenchymal stem cells-trophoblast cross-talk

**DOI:** 10.1371/journal.pone.0218437

**Published:** 2019-06-17

**Authors:** Daniela Surico, Valerio Bordino, Vincenzo Cantaluppi, David Mary, Sergio Gentilli, Alberto Oldani, Serena Farruggio, Carmela Melluzza, Giulia Raina, Elena Grossini

**Affiliations:** 1 Department of Translational Medicine, Gynecologic Unit, University East Piedmont, Azienda Ospedaliera Universitaria Maggiore della Carità, Novara, Italy; 2 Department of Translational Medicine, AGING PROJECT, University East Piedmont, Novara, Italy; 3 Department of Translational Medicine, Nephrology-Kidney Transplantation Unit and Center for Autoimmune and Allergic Diseases (CAAD), University East Piedmont, Azienda Ospedaliera Universitaria Maggiore della Carità, Novara, Italy; 4 Department of Translational Medicine, Laboratory of Physiology/Experimental Surgery, University East Piedmont, Novara, Italy; University of Insubria, ITALY

## Abstract

**Background:**

Oxidative stress is involved in the pathogenesis and maintenance of pregnancy-related disorders, such as intrauterine growth restriction (IUGR) and preeclampsia (PE). Human umbilical cord mesenchymal stem cells (hUMSCs) have been suggested as a possible therapeutic tool for the treatment of pregnancy-related disorders in view of their paracrine actions on trophoblast cells.

**Objectives:**

To quantify the plasma markers of peroxidation in patients affected by PE and IUGR and to examine the role of oxidative stress in the pathophysiology of PE and IUGR *in vitro* by using hUMSCs from physiological and pathological pregnancies and a trophoblast cell line (HTR-8/SVneo).

**Study design:**

In pathological and physiological pregnancies the plasma markers of oxidative stress, arterial blood pressure, serum uric acid, 24h proteinuria, weight gain and body mass index (BMI) were examined. Furthermore, the pulsatility index (PI) of uterine and umbilical arteries, and of fetal middle cerebral artery was measured. *In vitro*, the different responses of hUMSCs, taken from physiological and pathological pregnancies, and of HTR-8/SVneo to pregnancy-related hormones in terms of viability and nitric oxide (NO) release were investigated. In some experiments, the above measurements were performed on co-cultures between HTR-8/SVneo and hUMSCs.

**Results:**

The results obtained have shown that in pathological pregnancies, body mass index, serum acid uric, pulsatility index in uterine and umbilical arteries and markers of oxidative stress were higher than those found in physiological ones. Moreover, in PE and IUGR, a relation was observed between laboratory and clinical findings and the increased levels of oxidative stress. HTR-8/SVneo and hUMSCs showed reduced viability and increased NO production when stressed with H_2_O_2_. Finally, HTR-8/SVneo cultured in cross-talk with hUMSCs from pathological pregnancies showed a deterioration of cell viability and NO release when treated with pregnancy-related hormones.

**Conclusion:**

Our findings support that hUMSCs taken from patients affected by PE and IUGR have significant features in comparison with those from physiologic pregnancies. Moreover, the cross-talk between hUMSCs and trophoblast cells might be involved in the etiopathology of IUGR and PE secondary to oxidative stress.

## Introduction

During placentation, failure in remodeling of the spiral arteries by trophoblasts contributes to the development of pregnancy-related pathologies, such as preeclampsia (PE) and intrauterine growth restriction (IUGR) [1–4] via the excessive formation of reactive oxygen species (ROS) [4]. The subsequent peroxidation causes endothelial damage and changes in the release of pro-angiogenetic/vasodilating factors [5], which potentiate the endothelial dysfunction [6].

Nitric oxide (NO) can also play a fundamental role, being involved in blood flow regulation in the placental fetal vascular bed [7,8]. The balance of NO release by various NO synthases (NOS) could be at the basis of the onset of preeclamptic disorders [9,10]. An increase in systemic and uteroplacental NO, due to an overexpression of the inducible isoform of NOS [9] and an uncoupling of the endothelial NOS (eNOS), have been widely documented in animal models of PE [10]. It is important to underline the fact that an excess of NO can have deleterious rather than protective effects. In fact, an increased oxidative stress, which can be observed in pregnancies complicated by PE or IUGR, has been reported to be accompanied by an augmented iNOS expression and higher release of NO [11], which is converted to harmful peroxynitrite after reaction with superoxide [12].

Human umbilical cord mesenchymal stem cells (hUMSCs) could provide a valuable tool for the "rehabilitation" of dysfunctional trophoblast cells, allowing them to perform their essential roles for the development of the placenta through paracrine properties [13,14]. Hence, the injection of decidua-derived MSCs into preeclamptic mice was found to alleviate hypertension and proteinuria and to facilitate placental development [15]. Moreover, chorionic plate-derived MSCs were found to regulate trophoblast invasion and immune responses by inhibiting of proinflammatory cytokines like interleukin (IL)-1β, tumor necrosis factor (TNF)-α, interferon (IFN)-γ *in vitro* and *in vivo*[16].

Although the therapeutic potential of hUMSCs has been studied in various diseases [13], very little is known regarding possible protective effects elicited by hUMSCs isolated from physiologic pregnancies on trophoblast cells and changes in their cross-talk observed in peroxidative conditions or by using hUMSCs from PE or IUGR.

Therefore, this study examined the potential influence of hUMSCs collected from both physiological and pathological pregnancies on placental trophoblast cell line (HTR-8/SVneo). The results obtained have shown significant features of hUMSCs taken from pathologic pregnancies in terms of NO release and cell viability. Moreover, we have shown that an oxidative environment, such as the one observed in pathological pregnancies, can modulate the cross-talk between hUMSCs and trophoblast cell line.

## Materials and methods

### Clinical examination in patients

A total of 44 patients was recruited: 11 IUGR, 11 PE and 22 physiological pregnancies (healthy control) for this observational case-control study that was conducted at the Gynecology and Obstetrics Unit, University East Piedmont, Azienda Ospedaliera Universitaria (AOU) Maggiore della Carità, Novara, between November 2016 and June 2018. Participation in the study required written informed consent of patients and controls. The study was approved by the Intercompany Ethics Committee (Prot. n. 899/CE, study n° CE 143/16 approved on 21/11/2016) and complies with the Declaration of Helsinki and principles of Good Clinical Practice.

### Inclusion criteria

- Diagnosis of PE: blood pressure ≥140/90 mmHg in two measurements 6h apart + proteinuria (≥0.3 g/24 h, or urinary protein/creatinine ratio ≥30 mg/mmol)
- IUGR: fetus with an estimated fetal weight < 10th percentile by ultrasound [17] and growth arrest or pathologic PI of uterine arteries.
- Physiological pregnant women, aged-matched to patients.

### Exclusion criteria

- Age < 18 and > 46 years
- Previous diagnosis of hypertension, diabetes, autoimmune diseases, thrombotic microangiopathy, antiphospholipid antibody syndrome, chronic kidney disease (pre-pregnancy eGFR <60 mL/min/1.73m2)
- Treatment with anticoagulants
- Twin pregnancies
- Legal incapacity
- Smoking habit
- Infections (Fever, clinical symptoms of infection, Reactive C protein > 0,60 mg/100mL)

- Chromosomal aberrations
- PE women with concomitant IUGR diagnosis

### Clinical and ultrasound evaluation

All patients were subjected to careful anamnestic investigation. Risk factors were also investigated for pregnancy disorders and previous pathological pregnancies. In each patient, blood samples were collected in BD Vacutainer tubes containing EDTA or lithium/heparin for haemochrome and serum uric acid (performed at diagnosis) and lipid peroxide (performed at delivery) measurements. In addition, 24h proteinuria was measured in 24h urine samples taken in cases and controls at diagnosis.

Haemochrome, serum acid uric and 24h proteinuria were analyzed at the Clinical Chemistry and Biochemistry Unit, AOU Maggiore della Carità, Novara, by means of spectrophotometric methods (Sysmex XE 2100-Bellport NY and AGILENT LC/MS/MS1290+6410QQQ, Agilent, Santa Clara, CA, USA). Standardization, calibration of instruments and processing of samples were done according to manufacturer’s instructions. Samples were run in duplicate, and the average values were taken for each measurement.

For lipid peroxide measurement, plasma was placed in a refrigerator at 4° C for up to 24h and then transferred to the Physiology laboratory, University East Piedmont, AOU Maggiore della Carità, Novara, where quantifications were performed by expert biotechnologists blinded to the various conditions, as reported below. The same measurement was performed on umbilical cord blood samples.

### Ultrasound evaluation

The following ultrasound parameters were measured (Philips Affiniti 50 ultrasound system with a 6 MHz probe):

- Abdominal circumference (CA), cranial circumference (CC), CA/CC ratio;
- Amniotic fluid index (AFI);
- Flow of umbilical arteries (AAOO), the uterine arteries (UA), the middle cerebral arteries (MCA)
- Periodic monitoring of fetal growth [18].

The Doppler examination was performed by an expert gynecologist blinded to the physiological or pathological condition at maternal rest, in the absence of uterine contractions and in a slight lateral decubitus position [19].

In patients with physiological pregnancies, no other diagnostic or therapeutic treatments were used other than those indicated as “standard treatment” by the Ministry of Health guidelines [20].

### Velocimetry of UA, AAOO and MCA

The detection technique was based on the acquisition of a uterine parasagittal scan, with a slight lower and median inclination, up to the highlighting of the apparent intersection between the uterine artery and the external iliac artery. To evaluate the systolic-diastolic velocity gradient, the morphology of the velocimetry waveform was also analyzed. The persistence of the protodiastolic notch on both uterine velocimetric waves, beyond the 24th week, was considered pathological [21,22]. For flow measurements of the AAOO, the Doppler sample volume was placed in the vascular lumen encompassing it and the waveforms were recorded. The peak systolic and end-diastolic frequency shifts were then identified as previously described [23].

The MCA vessels were found with color or power Doppler ultrasound overlying the anterior wing of the sphenoid bone near the base of the skull [24].

### Data at delivery

In addition to duration of the dilating period and duration of the expulsive period, in newborns the following data were collected: Apgar score [25] at 1 and 5 min, gender and weight of the newborn, centile of weight according to the Ines Charts [26].

### Lipid peroxide assay

Patients/controls maternal blood and umbilical blood cords sample were centrifuged at 700–1,000 x g for 10 min at 4°C and plasma was transferred to a clean tube and stored on ice. Thiobarbituric reactive substances (TBARS) assay, that measures malonyldialdeide (MDA) in samples, was performed as previously described [27] by using the TBARS assay (Cayman Chemical, Michigan, USA). The absorbance of supernatants was read at 530–540 nm, using a spectrometer (BS1000 Spectra Count, San Jose, CA, USA).

### Isolation and propagation of hUCMSCs

Processing of the umbilical cords and the isolation of the hUCMSCs were carried out at the Physiology laboratory, by expert biotechnologists, as previously described [28]. Briefly, umbilical cord Wharton’s jelly and amniotic membranes were rinsed with phosphate buffer solution (PBS) several times and dissected into short pieces. Then, the small pieces were transferred into T-75 flasks containing 10 mL Dulbecco’s Modified Eagle Medium (DMEM)/F12 (Euroclone, Pero, Milan, Italy) with the addition of 10% foetal bovine serum (FBS; Sigma, Milan, Italy) and 1% penicillin/streptomycin (P/S; Sigma) and incubated at 37°C with 5% CO2. The remnants of the cord fragments were removed after 7–10 days of culture, and the cultures were fed every 3 days thereafter. At confluence, cells were trypsinized and passed into new flasks for further expansion [29]. For each pathological condition and for control group, isolated hUCMSCs were grouped into three different pools for practical reasons related to the management of cells and the performing of experiments.

### Detection of hUCMSCs surface markers

After three passages, hUCMSCs were examined for surface markers expression. Cells were trypsinized and washed with and resuspended in PBS to a density of 1×10^6^ cells/mL. A volume of 100 𝜇L of this cell suspension was added to 1.5mL Eppendorf tubes. Tube 1 was used as a negative control (with PBS), and the other tubes were incubated with isotype control-FITC and CD105-FITC (BD Biosciences, Milan, Italy) antibodies for 30 min. The cells were then analyzed using flow cytometry.

### *In vitro* experiments

Experiments on hUCMSCs and HTR-8/SVneo cell line (ATCC, LGC Standards S.r.l., Sesto San Giovanni, MI Italy)[30], were carried out at the Physiology laboratory, by expert biotechnologists blinded to various physiologic/pathologic conditions.

### HTR-8/SVneo cell line

The HTR-8/SVneo cell line was derived by transfecting the cells that grew out of chorionic villi explants of human first-trimester placenta and immortalized by the simian virus 40 large T antigen. These cells exhibit a variety of markers characteristic of extravillous invasive trophoblast cells *in situ* and are useful to study trophoblast and placental biology [31,32].

### Cell culture

hUMSCs were cultured in T-75 flasks containing 10 mL DMEM/F12 for 24h, whereas HTR-8/SVneo cells were grown in Roswell Park Memorial Institute (RPMI) medium (Euroclone) (DMEM and RPMI with 10% FBS, 1% glutamine and 1% P/S). hUMSCs and HTR-8/SVneo cells grown alone were stimulated with human chorionic gonadotropin (100 μM and 100 nM hCG; Sigma) and 17β-estradiol (1 nM and 100 pM; Sigma) for 30 min, in either physiological condition or oxidative stress condition induced by 30 min 200 μM hydrogen peroxide given after 30 min pre-treatment with the above hormones [33,34]. The used concentrations of hCG and estradiol were in the range of plasma values found in humans [35,36]. Also hydrogen peroxide was used at a concentration similar to the one previously used in the same cell lines [37]. The culture medium from hUMSCs was also collected and used for co-stimulation experiments after centrifugation and filtration. In co-stimulation experiments, HTR-8/SVneo cells were treated for 24h with supernatants of hUMSCs. The production of NO and cell viability were examined, as reported below.

### NO release

The NO production was measured in culture supernatants by using the Griess method (Promega, Milan, Italy), as previously performed [33,38–40]. At the end of incubation, the absorbance at 570 nm was measured by a spectrometer (BS1000 Spectra Count) and the NO production was quantified in respect of nitrite standard curve and expressed as μmol.

### Cell viability

As described for NO release, cell viability of hUMSCs and HTR-8/SVneo cells was examined by using the 1% 3-[4,5-dimethylthiazol-2-yl]-2,5-diphenyl tetrazolium bromide (MTT; Life Technologies Italia, Monza, Italy) dye, as previously described [40]. After each treatment, cell viability was determined by measuring the absorbance (620 nm) through a spectrometer (BS1000 Spectra Count) [33,38,39].

### Statistical analysis

Statistical analysis was performed using the STATVIEW version 5.0.1 for Microsoft Windows (SAS Institute Inc., Cary NC, USA). For the nominal data, the exact Fisher test was used. For the correlations, the Pearson’s correlation coefficient was calculated. Data from *in vitro* study were checked for normality before statistical analysis The "One-Way ANOVA" test followed by the *post-hoc* Bonferroni test were used to examine changes between the different experimental conditions. The Mann Whitney U test was used to compare percentage values. Data are expressed as mean ± SD (standard deviation). A value of p<0.05 was considered statistically significant.

## Results

### Clinical variables

**Fig 1:**
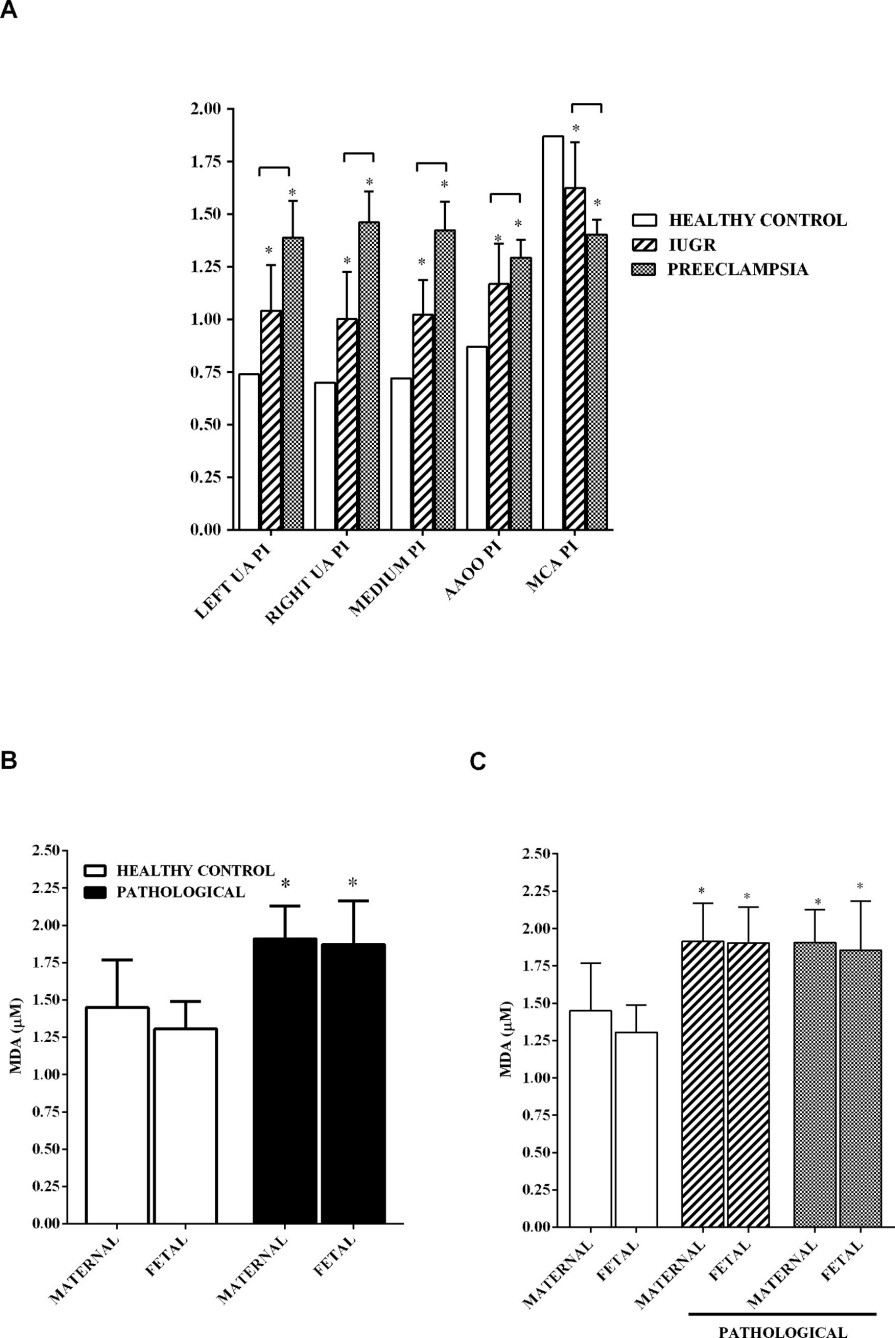
Doppler velocimetry and oxidative stress in maternal/fetal blood. (**A)** UA: uterine artery; AAOO: umbilical artery; MCA: middle cerebral artery; (**B)** comparison between oxidative stress condition measured in healthy controls and pathological groups. (**C)** stratification of patients based on pathology. IUGR: intrauterine growth restriction; MDA: malonyldialdeide (μM). The values of MDA represent the means ± SD of three different measurements. Square brackets indicate significance between groups. *p<0.05 vs healthy control.

Anthropometric and clinical variables of patients and controls are reported in Table 1. Patients with pregnancy-related disorders had a higher body mass index (BMI) in comparison with controls, although the difference was significant only in the IUGR group (p <0.05). The systolic and diastolic blood pressure, uricemia and 24h proteinuria were also significantly higher in PE patients compared to both controls and IUGR group (Table 1; p <0.05). AC was significantly lower in fetuses of IUGR and PE patients (p <0.05), whereas PI values in UA and AAOO were greater than reference values of healthy controls [41] (Table 1 and Fig 1A; p <0.05). Moreover, in the PE group, the pulsatility index in UA and AAOO was higher than the one found in the IUGR group (p <0.05). The PI of MCA was significantly lower in the pathological groups compared to reference values (p <0.05). Moreover, the PE group showed significantly reduced MCA PI compared to the IUGR group (Table 1 and Fig 1A; p<0.05). As shown in Fig 1B and 1C, a higher MDA level was detected in both maternal and fetal plasma of pathological samples compared to control ones (p <0.05).

**Table 1:** Clinical, laboratory and Doppler velocimetric parameters.

	CONTROLS (n = 22)	IUGR (n = 11)	PE (n = 11)
**PARAMETER**			
Age (years)	33.45 ± 5.03	35.18 ± 6.52	33.83 ± 4.97
Italian patients	16 (73%)	7 (64%)	5 (45%)
Foreign patients	6 (27%)	4 (36%)	6 (55%)
BMI (Body Mass Index)	21.77 ± 2.58	25.35 ±3.65 *	23.75 ± 5
Weight gain in pregnancy	10.91 ± 3.60	8.09 ± 7.13	13 ± 4.02 ^†^
Gestational age at delivery (days)	277.82 ± 7.21	259.27 ± 16.56 *	240.90 ± 22.01 *^†^
Systolic Blood Pressure (mmHg)	115 ± 12.22	121 ± 12	135 ± 10*^†^
Diastolic Blood Pressure (mmHg)	69 ± 9.06	72 ± 10	85 ± 10 *^†^
Primiparous patients	13 (59%)	7 (64%)	9 (82%)
Pluriparous patients	9 (41%)	4 (36%)	2 (18%)
Serum uric acid (mg/dl)	4.04 ± 1.22	4.63 ± 1.56	6.33 ± 1.25 *^†^
Proteinuria mg/24h	6.36 ± 10.02	52.13 ± 84.48	2638.15 ± 2628.08*^†^
PI L UA	0.74 §	1.041 ± 0.161 *	1.39 ± 0.16 *^†^
PI R UA	0.70 §	1.00 ± 0.21 *	1.46 ± 0.14 *^†^
Medium PI UA	0.72 §	1.02 ± 0.14 *	1.42 ± 0.13 *^†^
PI AAOO	0.87 §	1.17 ± 0.182 *	1.29 ± 0.08 *^†^
PI MCA	1.87 §	1.62 ± 0.03 *^‡^	1.41 ± 0.06 *
Fetal Gender	M = 11 (50%); F = 11 (50%)	M = 8 (73%); F = 3 (27%)	M = 5 (45%); F = 6 (55%)
Centile of birthweight	41.82 ± 20.40	6.63 ± 5.63 *^‡^	34.58 ± 28.38
Apgar 1'	8.73 ± 0.63	8.18 ± 1.54	8.00 ± 0.85 *
Apgar 5'	10	8.82 ± 0.60 *	8.67 ± 0.49 *
AC Centile	54.17 ± 17.17	4.00 ± 1.18 *^‡^	30.00 ± 30.21 *
Spontaneous Delivery	19 (86%)	3 (27%)*	0 (0%)*
Induced delivery	0 (0%)	4 (36.5%) *	4 (36%) *
Caesarean delivery	3 (14%)	4 (36.5%) *	7 (64%) *

AC: abdominal circumference; PE: preeclampsia; IUGR: intrauterine growth restriction; PI L UA: pulsatility index left uterine artery; PI R UA: pulsatility index right uterine artery; Medium PI UA; average PI of uterine arteries; MCA: middle cerebral artery; AAOO: umbilical artery.

* = p<0.05 vs controls

‡ = p<0.05 vs PE

† = p<0.05 vs IUGR

§: literature references.

**Fig 2:**
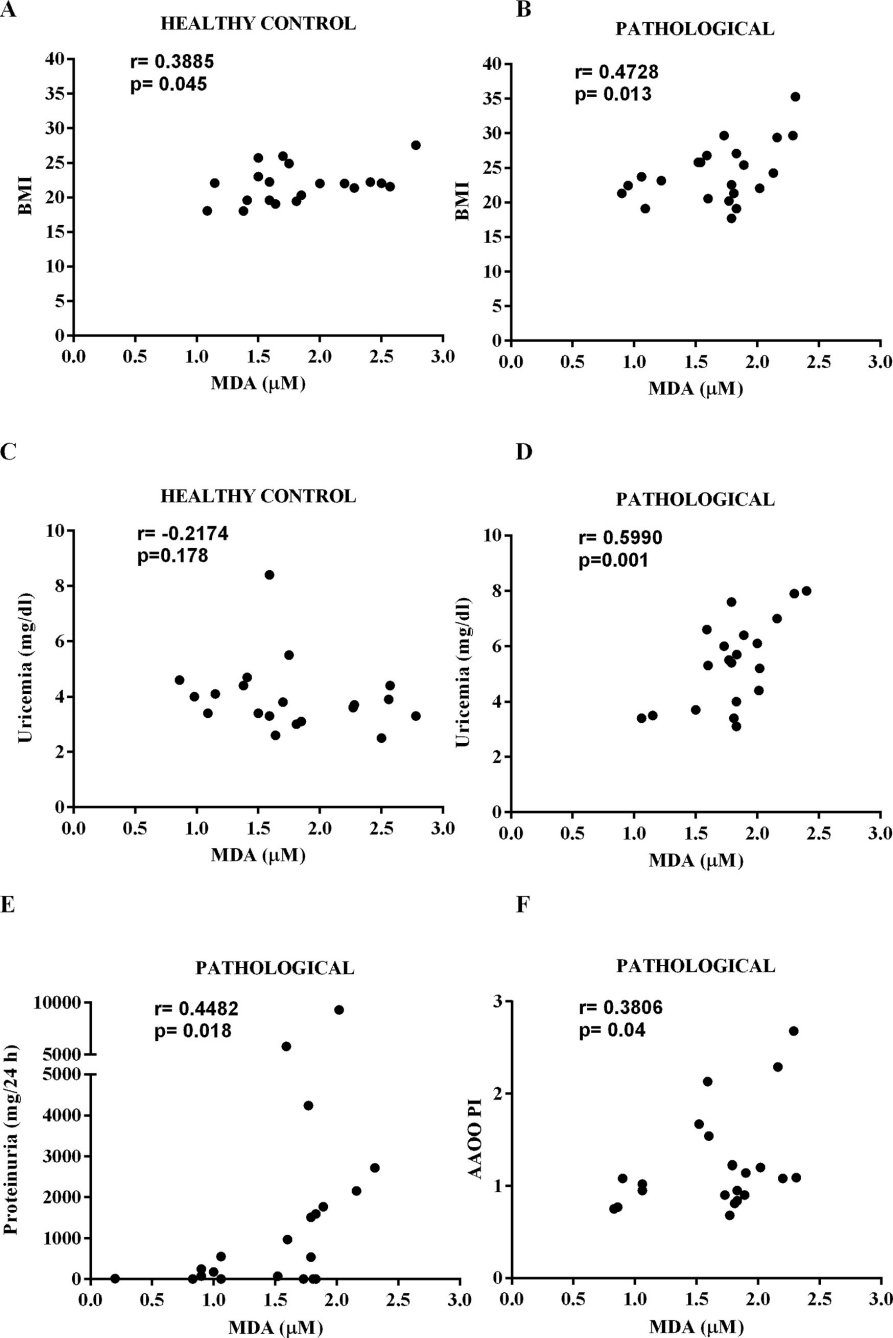
Correlation between different clinical parameters and MDA (μM) in control and pathological groups. BMI: body mass index. Other abbreviations are as in Fig 1.

Furthermore, BMI, uricemia, 24h proteinuria and the PI of AAOO were positively correlated with MDA of patients affected by pregnancy-related pathologies (Fig 2).

### *In vitro* studies

hUCMSCs reached ∼80% confluency after 2 weeks and were then trypsinized and passaged at a density of 10x10^4^ cells/mL. Moreover, the 84% of total isolated cells was positive to the CD105 surface marker, which confirmed that they were blood cord mesenchymal cells [42].

### Effects of hCG and 17β-estradiol on NO release and cell viability of hUMSCs

**Fig 3:**
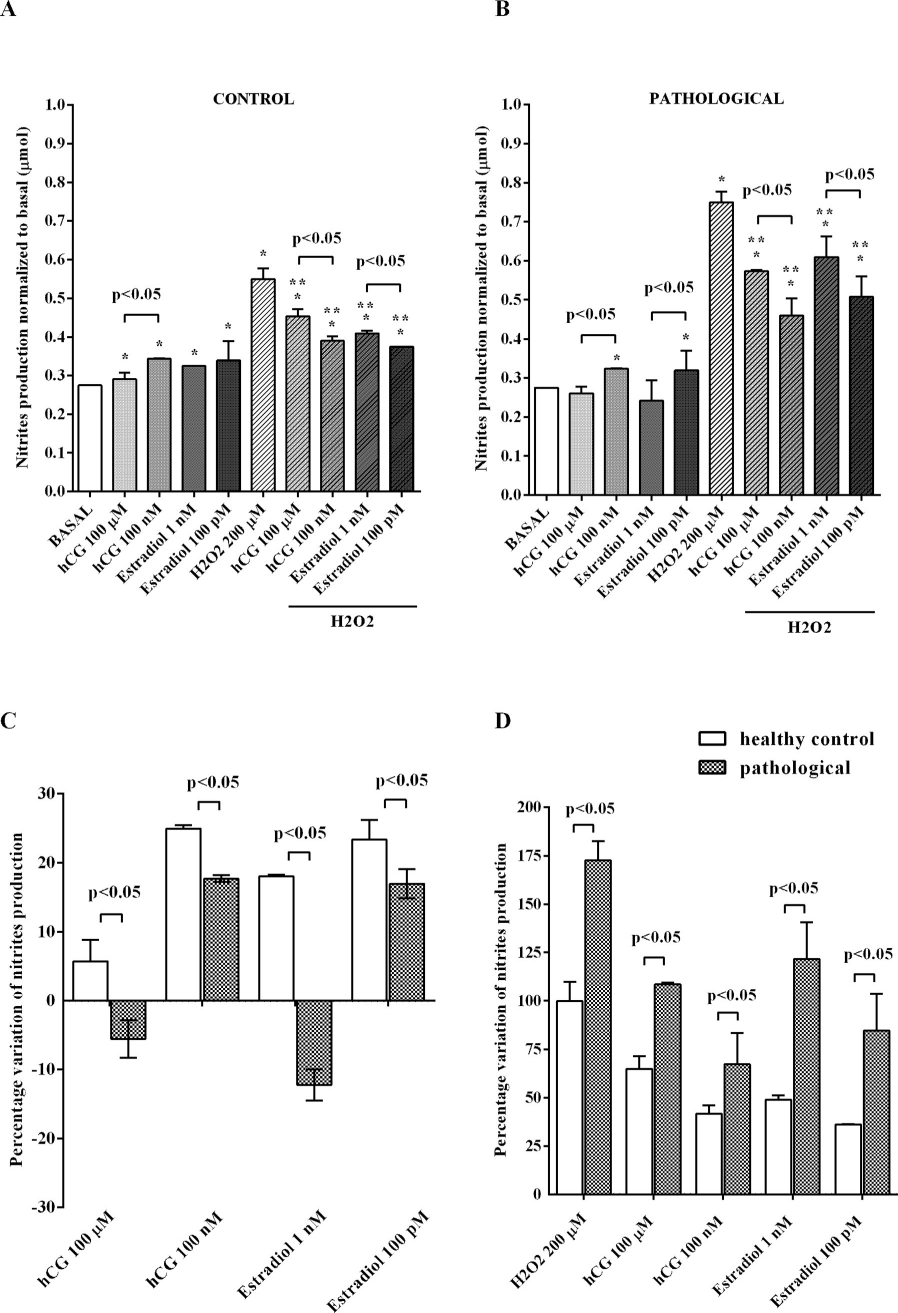
Effects of hCG and 17β-estradiol on NO release in hUMSCs cultured in physiological or peroxidative conditions. **(A)** hUMSCs extracted from normal umbilical cords and mixed into three different pools; (**B)** hUMSCs extracted from pathological umbilical cords and mixed into three different pools. (**C)** Comparison between the variations of NO release in physiological conditions by hUMSCs extracted from normal and pathological umbilical cords and mixed into three different pools for both physiologic and pathologic conditions. (**D)** Comparison between the variations of NO release in oxidative stress conditions by hUMSCs extracted from normal and pathological umbilical cords and mixed into three different pools. hCG: human chorionic gonadotropin. The values obtained correspond to the NO (μmol) produced, after each stimulation, by samples containing 1.5 μg of proteins each. They are expressed as normalized to control values (Basal). Basal: cells non-treated. Reported data are means ± SD of five independent experiments. Square brackets indicate significance between groups. *p<0.05 vs BASAL; **p<0.05 vs H2O2 200μM.

**Fig 4:**
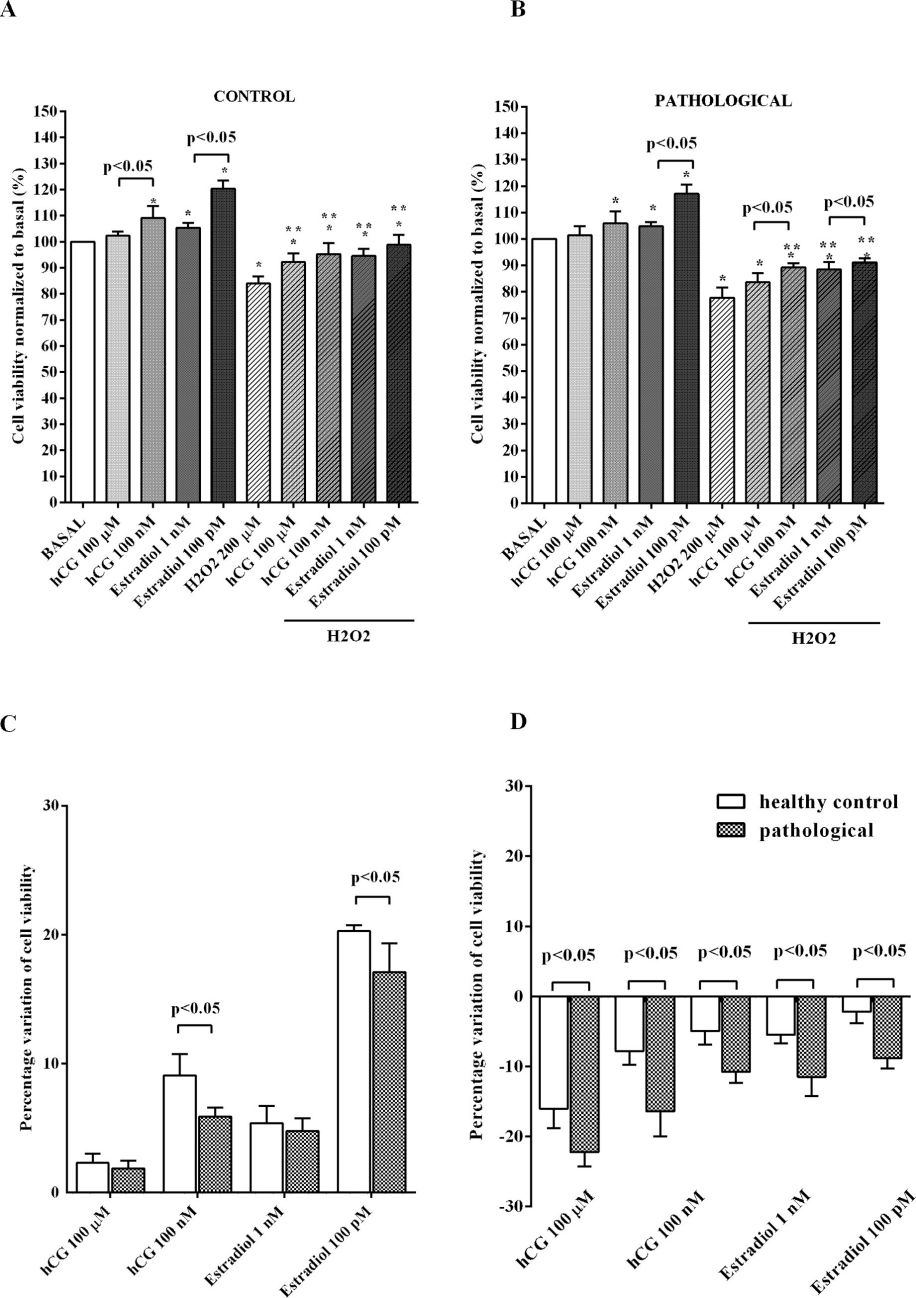
Effects of hCG and 17β-estradiol on cell viability of hUMSCs cultured in physiological or peroxidative conditions. **(A)** hUMSCs extracted from normal umbilical cords and mixed into three different pools; (**B)** hUMSCs extracted from pathological umbilical cords and mixed into three different pools. (**C)** Comparison between the variations of cell viability in physiological conditions of hUMSCs extracted from normal and pathological umbilical cords and mixed into three different pools. (**D)** Comparison between the variations of cell viability in physiological conditions of hUMSCs extracted from normal and pathological umbilical cords and mixed into three different pools. Abbreviations are as in Fig 5. They are expressed as percentage normalized to control values (Basal). Reported data are means ± SD of five independent experiments. Square brackets indicate significance between groups. *p<0.05 vs BASAL; **p<0.05 vs H2O2 200μM.

Both hCG and 17β-estradiol increased NO release and cell viability in physiological conditions. Regarding cell viability, our findings on the effects of estradiol confirmed previous ones obtained in the same cell line [43]. Also the effects of 100 nM hCG and 100 pM 17β-estradiol on NO release in hUMSCs isolated from pathological cords were lower than those observed in hUMSCs isolated from normal umbilical cords, as shown in Fig 3. It can be observed that the percentage variation of NO release induced by 100 nM and 100 μM hCG in hUMSCs amounted to 24.9 ± 0.5 and 5.7 ± 3, respectively, in physiologic conditions, and to 17.7 ± 0.5 and -5.5 ± 2.7, respectively, in pathologic conditions. As regarding estradiol, the percentage variation of NO release induced by 100 pM and 1 nM 17β-estradiol in hUMSCs amounted to 23.4 ± 2.8 and 18 ± 0.2, respectively, in physiologic conditions, and to 16.9 ± 2.1 and -12.2 ± 2.2, respectively, in pathologic conditions. The differences observed between the physiologic and pathologic conditions were statistically significant (p<0.05). Hydrogen peroxide increased NO release by 99.8% ± 9.5 of basal levels in hUMSCs isolated from normal umbilical cords and 172.6% ± 9.9 of basal levels in hUMSCs isolated from pathologic umbilical cords (Fig 3D). NO release caused by hydrogen peroxide in hUMSCs isolated from pathological umbilical cords was 73.1% ± 9.1 higher than that found in hUMSCs isolated from physiologic umbilical cords (p<0.05). Moreover, it reduced cell viability to about 22.2% ± 2 of basal levels in hUMSCs isolated from pathological umbilical cords and to 16% ± 2.7 of basal levels in hUMSCs isolated from normal umbilical cords (Fig 4B and 4D). Cell viability of hUMSCs from pathological umbilical cords treated with hydrogen peroxide was 40.9% ± 18.9 lower than that of hUMSCs isolated from physiologic umbilical cords (p<0.05). The pre-treatment with hCG and 17β-estradiol, reduced the deleterious effects of oxidative stimuli in hUMSCs, particularly in cells taken from healthy control (Fig 3C and 3D; Fig 4C and 4D).

### Effects of hCG and 17β-estradiol on NO release and cell viability of HTR-8/SVneo cells

**Fig 5:**
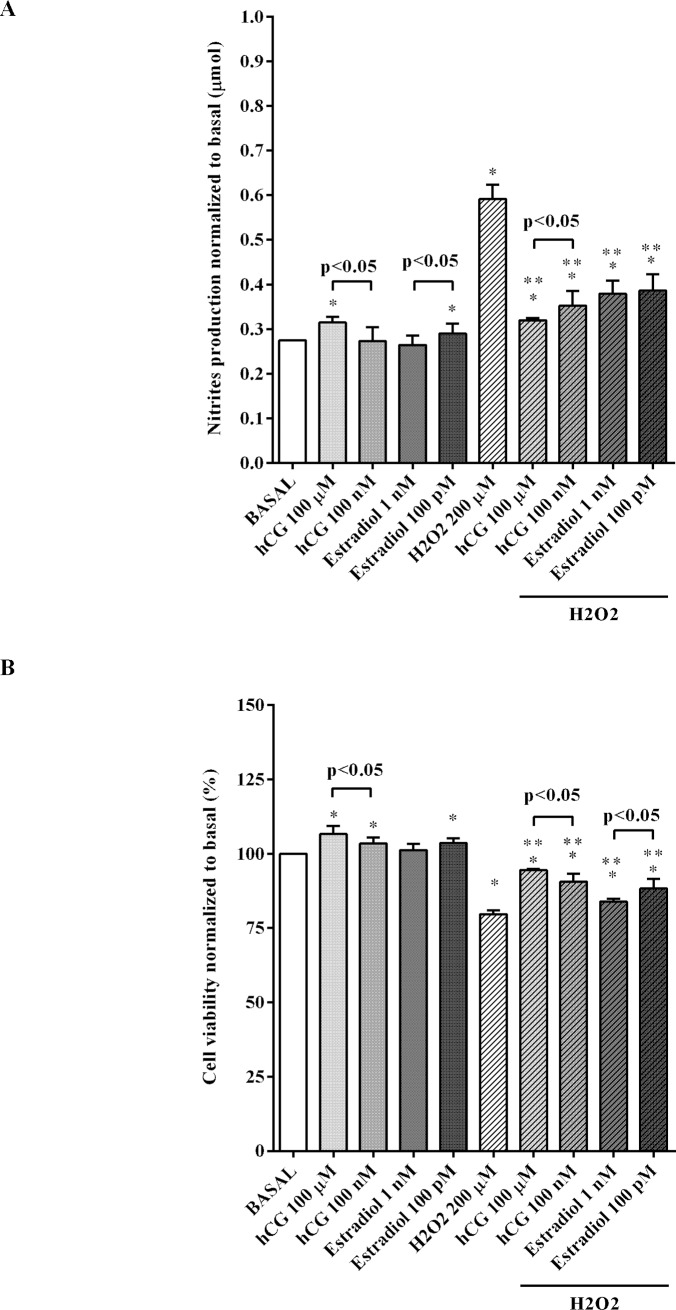
**Effects of hCG and 17β-estradiol on NO production (A) and cell viability (B) in HTR-8/SVneo cells cultured in physiological or peroxidative conditions.** Abbreviations are as in previous Figs. Data are expressed as normalized to Basal. Reported data are means ± SD of five independent experiments. Square brackets indicate significance between groups. *p<0.05 vs BASAL; **p<0.05 vs H2O2 200μM.

Only treatment for 30 min with 100 μM hGC and 100 pM 17β-estradiol were able to increase the production of NO by trophoblast cells in physiological conditions (Fig 5A, p<0.05). In addition, both hormones counteracted the effects of hydrogen peroxide at 100 μM hGC and 100 pM (Fig 5A). As shown in Fig 5B, cell viability of trophoblast cells treated with 100 nM and 100 μM hGC and with 100 pM 17β-estradiol was significantly higher than that observed in basal levels (p<0.05). Oxidative stress strongly reduced cell viability of HTR-8/SV neo cells, an effect which was counteracted by both hormones at all doses (p<0.05).

### Cross-talk between hUMSCs and HTR-8/SVneo cells

**Fig 6:**
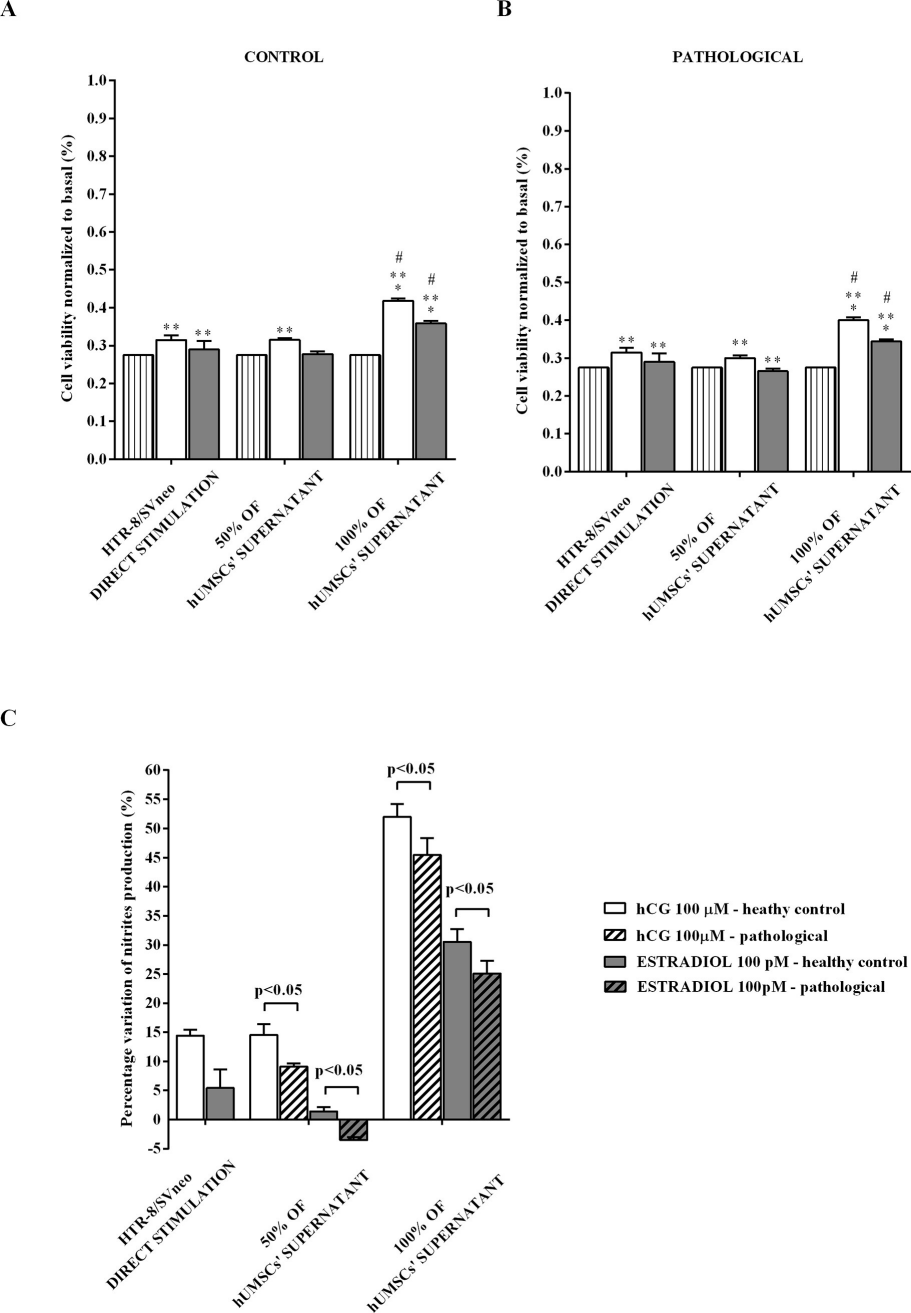
Cross-talk between hUMSCs and HTR-8/SVneo cells: Effects on NO release. (**A)** HTR-8/SVneo cells were cultured for 24 h in a medium containing 0% (HTR-8/SVneo cells directly stimulated with hormones), 50%, or 100% hUMSCs’ supernatants taken from healthy umbilical cords. (**B)** HTR-8/SVneo cells were cultured for 24 h in a medium containing 0% (HTR-8/SVneo cells directly stimulated with hormones), 50%, or 100% hUMSCs’ supernatants taken from pathological umbilical cords. (**C)** comparison between the percentage variations in NO release by HTR-8/SVneo cells stimulated with medium containing 0%, 50% and 100% supernatant of hUMSCs from normal and pathological umbilical cords. The values obtained correspond to the NO (μmol) produced, after each stimulation, by samples containing 1.5 μg of proteins each. They are expressed as normalized to Basal. Reported data are means ± SD of five independent experiments. Square brackets indicate significance between groups. *p<0.05 vs HTR-8/SVneo direct stimulation; **p<0.05 vs BASAL; ^#^ vs 50% of hUMSCs’ supernatants.

**Fig 7:**
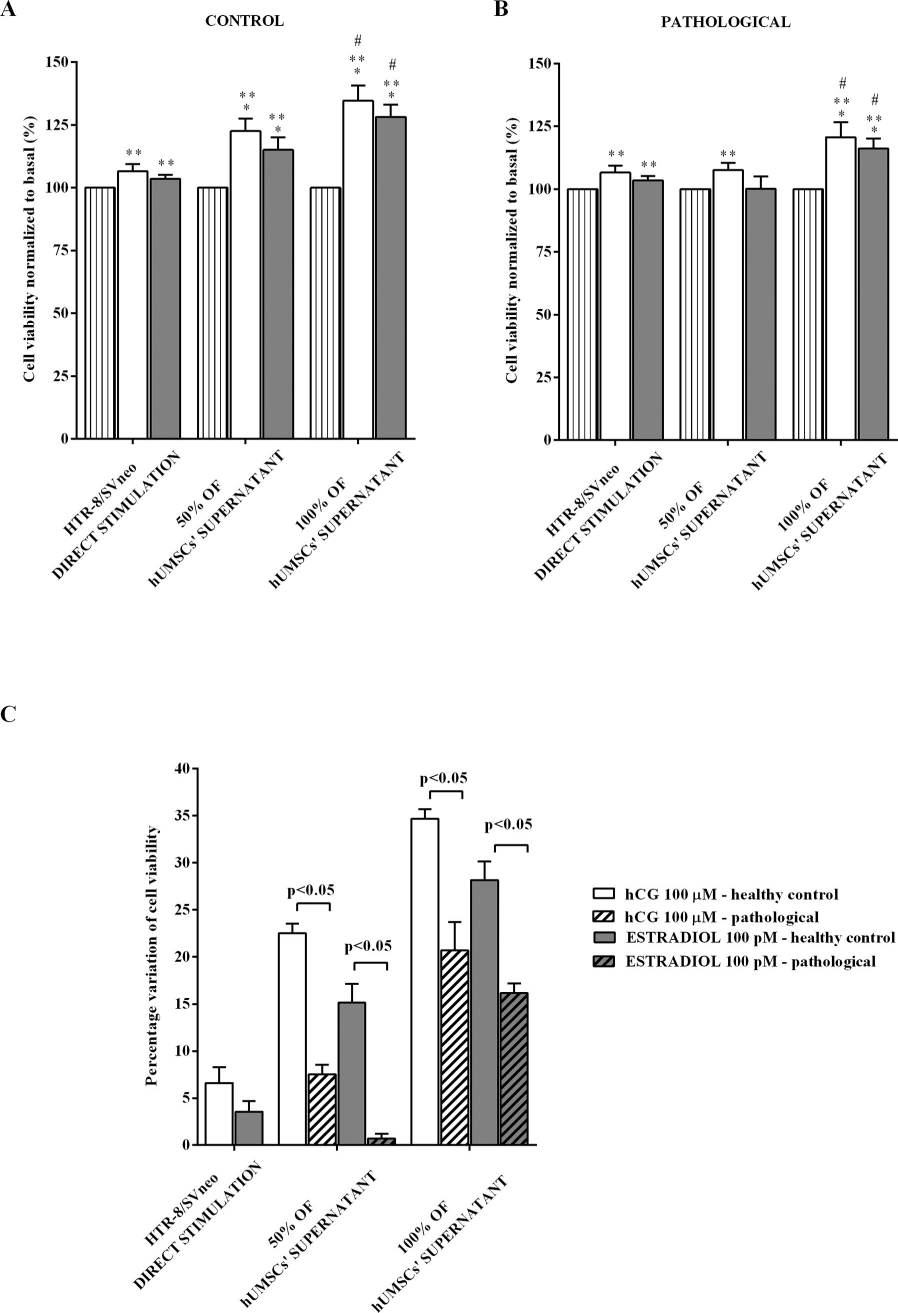
Cross-talk between hUMSCs and HTR-8/SVneo cells: Effects on cell viability. (**A)** HTR-8/SVneo cells were cultured for 24 h in a medium containing 0% (HTR-8/SVneo cells directly stimulated with hormones), 50%, or 100% hUMSCs’ supernatants taken from healthy umbilical cords. (**B)** HTR-8/SVneo cells were cultured for 24 h in a medium containing 0% (HTR-8/SVneo cells directly stimulated with hormones), 50%, or 100% hUMSCs’ supernatants taken from pathological umbilical cords. (**C)** Comparison between the variations of cell viability of HTR-8/SVneo cells cultured with medium containing 0%, 50%, or 100% hUMSCs’ supernatants taken from normal and pathological umbilical cords. They are expressed as percentage normalized to control values (Basal). Reported data are means ± SD of five independent experiments. Square brackets indicate significance between groups. *p<0.05 vs 0% HTR-8/SVneo direct stimulation; **p<0.05 vs BASAL; ^#^ vs 50% of hUMSCs’ supernatants.

HTR-8/SVneo cells were incubated, as described in Materials and Methods, with 0% (HTR-8/SVneo trophoblast cells directly stimulated with 100 μM hGC and 100 pM 17β-estradiol in standard culture medium), 50%, or 100% supernatant of hUMSCs. As shown in Fig 6, NO release by HTR-8/SVneo cells was greater in the presence of 100% supernatant than 50% supernatant or 0% supernatant (p<0.05). In addition, the response was higher when the supernatants were taken from hUMSCs of healthy controls (p<0.05). Moreover, as shown in Fig 7, in HTR-8/SVneo cells treated with supernatants taken from hUMSCs a significantly higher cell viability was observed in comparison with that one found in cells cultured only with standard culture medium. This effect was related to the dilution of the supernatants and was greater in the trophoblast cells cultured with supernatants taken from hUMSCs isolated from healthy control.

## Discussion

The results obtained in the present study showed that a “peroxidative” environment, such as the one that characterizes PE or IUGR, can modulate the viability and NO release of hUMSCs and HTR-8/SVneo cell line under exposure to” pregnancy-related hormones,”potentially through changes of their cross-talk.

Although advances in obstetric techniques allow identification and monitoring of complex pregnancy-related pathologies, such as IUGR and PE, in-depth knowledge about the molecular mechanisms underlying their onset and maintenance is still scarce. Currently the only therapeutic intervention applicable is to induce delivery, which potentially involves an increased fetal (prematurity) and maternal (procedure of caesarean section) risk for morbidity. The most deficient area in terms of knowledge and effective interventions is prevention.

In our study, patients with pregnancy-related disorders showed a higher BMI in comparison with controls. Regarding weight gain during pregnancy, PE group showed increased values compared with IUGR group. In this regard, it should be recalled that overweight, obesity and weight gain during pregnancy, in the absence of co-factors such as diabetes and hypothyroidism, have been widely considered as independent risk factors for the development of pregnancy diseases, especially PE [44].

Regarding the laboratory data, we showed significantly increased levels of serum uric acid in PE patients compared to healthy controls and IUGR group. This is consistent with previously published data about the role of uric acid in the diagnosis and pathogenesis of PE [45]. Interestingly, uric acid has recently been found to act as cofactor in endothelial damage and erroneous maternal vascular remodeling [46]. Hence, through interaction with NACHT, LRR and PYD domains-containing protein 3 (NLRP3) inflammasome, uric acid could contribute to the induction of endothelial inflammation by increasing IL-1β levels [47].

As for fetal biometric ultrasound data, we considered the differences of AC between the pathological groups and controls. In order to compare the values in relation to the different gestational ages, the AC centiles were used [48]. Both the IUGR and PE groups reported lower AC values compared to healthy controls, which was a sign of altered fetal growth in utero. Moreover, in our sample, significantly elevated levels of PI in the UA of the PE and IUGR groups were found, as has been previously reported [49]. Also regarding the PI of the UA, we have shown higher indexes in the IUGR and PE groups compared to the reference values, indicating an increase in vascular resistance also in the fetal umbilical arteries, as has previously been shown [50]. The significant reduction of the MCA PI observed in both pathological groups could indicate the implementation by the fetus of a brain sparing mechanism, which consists of the reduction of cerebral vascular resistance in response to tissue hypoxia caused by reduced blood supply. The greater the compensation mechanism, the worse the clinical picture and the predictable fetal outcome (short and long term) [51]. According to our results, therefore, the PE group showed fetuses with a more impaired perfusion than the IUGR group, being most evident the brain-sparing mechanism.

Oxidative stress has widely been considered as playing a role in the pathophysiology of pregnancy-related disorders [44]. By evaluating the differences in oxidative stress in maternal and fetal plasma between physiological and pathological groups, we found increased MDA levels in samples taken from patients with pregnancy-related disorders, in agreement with the literature [44]. Those levels were positively correlated with BMI, uricemia and 24 h proteinuria in pathologic groups.

Also the positive correlation between PI in the UA and MDA levels indicated a close relationship between oxidative stress and the increase of vascular resistance on the fetal side [50]. In this context, it should be remembered that oxidative stress could affect perfusion by alteration the endothelial function [52] and NO release. Hence, it is important to highlight that NO is crucial for endovascular invasion induced by trophoblasts during pregnancy [53,54]. If the process fails, the utero-placental vasculature changes from a low resistance to a high resistance condition with a resulting hypoperfusion. As a consequence, the placental ischemic status could maintain the endothelial damage [1,12,55].

As previously shown, an altered production of NO might represent an important pathogenetic mechanism underlying placental pathologies [8]. In particular, a strong increase in utero-placental NO [9,56] could have deleterious rather than protective effects, as a consequence of its conversion into peroxinitrites.

The results obtained from the *in vitro* experiments showed that, under physiological condition, hUMSCs and trophoblasts cells increased cell viability in response to hCG and 17β-estradiol, which were used at concentrations comparable to those found *in vivo* [35,36], and were protected against the deleterious effects of hydrogen peroxide.

It is to note that HTR-8/SVneo cell line shares many markers of extravillous invasive trophoblast cells. Thus, previous *in vitro* data about the cross-talk of HTR-8/SVneo and healthy hUMSCs was related to the influence of migratory and invasive abilities of the former [29]. We have chosen HTR-8/SVneo cell line basing on the role of early pregnancy extravillous cytotrophoblasts on spiral artery remodeling [57] and its relation to the generation of oxidative stress [58,59].Our study is the first to examine the cross-talk HTR-8/SVneo and hUMSCs from pathologic pregnancies with regard to cell viability under gestational endocrine stimuli.

The peroxidative environment was simulated in our experiments by using hydrogen peroxide at similar concentrations as those previously used in the same cell lines [37,60], since it is physiologically produced in maternal circulation and its levels are increased in preeclamptic patients in the early stages of pregnancy [61]. Also, the concentration of hydrogen peroxide was similar to the one that has been shown to cause senescence in hUSMCs [60].

Regarding the NO release, an increase in both hUMSCs and trophoblast cells was also found. Our findings about the existence in HTR-8/SVneo of a physiological NO production measured by using the Griess assay are in agreement with previous ones obtained in the same cell line by using the 4-amino-5-methylamino-2′,7′-difluorofluorescein diacetate system for NO measurement [57].

In contrast, in cells treated with hydrogen peroxide, the release of NO has been counteracted by both hormones. Furthermore, hCG and 17β-estradiol were less effective in modulating NO release and keeping cell viability on hUMSCs isolated from pathological pregnancies.

The intracellular signaling downstream estrogenic receptors that has been proven to have a critical role in estradiol action as a survival agent, includes p38 mitogen activated protein kinases (MAPK) and Akt/phosphatase and ERK activation [34]. The above kinases have been found to play a role in exerting the effects of hCG on NO release and cell viability, as well [33,62]. In both cases, the involvement of various NOS isoforms and changes of mitochondrial function observed in physiological or peroxidative conditions would be the final target downstream the above pathways activation [33,34].

The different involvement of pathways that are NO-related could also explain the absence of effects of sildenafil for severe, early-onset fetal growth restriction in terms of prolongation of pregnancy, neonatal survival, morbidity, or feto-placental perfusion [63].

Thus, our data about changes of NO release by hUMSCs isolated from physiological or pathological pregnancies and by trophoblast cells cultured in either physiological or oxidative stress conditions in response to hCG and estradiol, could represent a physio-pathological mechanism at the basis of the altered utero-vascular perfusion observed in patients.

Also, the results obtained with co-stimulation experiments showed a different pattern of cross reaction exerted by hUMSCs on trophoblast cells depending on the physiological or pathological conditions. Hence, both cell viability of HRT-8/ SVneo cells and NO release were differently affected by supernatants of hUMSCs taken from normal or pathological cords. It is also well known that hUMSCs can regulate trophoblast invasion and the immune response through their immuno-modulatory effects and the release of vascular endothelial growth factor (VEGF) [64], the placental growth factor [12], microvesicles [65], NO, or miRNAs [66–68].

In particular, recent evidence has suggested an important role of epigenetic changes in the pathogenesis and maintenance of PE and IUGR. Thus, a crucial role is covered by miRNA, whose expression strongly changes in normal and pathological pregnancies [66–68]. Also, miR‑335 and miR‑584 have been found to act as regulator of HTR8/Svneo cells migration and invasion by targeting NO release [69].

Our results confirm previous data regarding the importance of the cross-talk between hUMSCs and trophoblast cells for the proper functioning of the latter [29,70]. About this issue it is noteworthy that in the animal models of PE, the infusion of blood cord stem cells taken from patients with physiological pregnancies improved the systemic pathological condition and caused an increase of the fetal weight [70]. There is only one reported clinical study performed on a small number of patients, which demonstrated a potential positive role of hUMSCs supernatant on HTR-8/SVneo proliferation, migration, invasion, and secretion function [29]. However, those patients had given birth at the end of physiological pregnancies by caesarean section. In our study we have expanded the number of patients and used cells from physiological and pathologic pregnancies.

So far, it remains to be determined how our observed *in vitro* effects on hUMSCs are transmitted to the extravillous compartment *in vivo* (e.g. indirectly via interposition of syncytiotrophoblast or villous cytotrophoblasts or as a direct endocrine effect).

Our findings about the role of oxidative stress in PE and IUGR could have clinical implications regarding the usefulness of preventive or therapeutic treatments aimed at contrasting oxidative stress [71], such as the introduction of antioxidant elements in the diet of patients with risk factors for PE and IUGR or already diagnosed patients. Furthermore, the important role of oxidative stress in pregnancy-related disorders may allow the identification of a possible early marker of disease risk, to start monitoring the patient at early stages of pregnancy [72]. Among various factors, endothelial progenitor cells and NK cells in peripheral blood during the first trimester could be considered a valuable tool for the early identification of women at risk of developing PE[73]. Also serum neurokinin-B, whose levels were found to be higher in the normotensive pregnant females in comparison with the preeclamptic females, could represent an early marker of disease [74].

Our results might further have pathophysiologic implications for endometriosis patients, a condition characterized by increased oxidative stress and a higher incidence of PE and IUGR [75].

Additional research is, however, required to define an appropriate early diagnostic method and treatment to counteract the oxidative stress involved in the pathogenesis and maintenance of those diseases. Moreover, it would be very interesting to analyze which are the molecules actually involved in the cross-talk between hUMSCs and trophoblast cells, which may provide a valuable tool for remediating dysfunctional trophoblast cells [29].

In conclusion, the results we have obtained have highlighted the role of oxidative stress in the pathogenesis and maintenance of IUGR and PE. Moreover, we have shown how an environment characterized by oxidative stress, as the one of hUMSCs taken from pathological patients, could negatively affect their behavior and their cross-talk with trophoblast cells.

### Main limitations of this study

Our study is limited by significant differences in gestational age and birth modes between control and PE, IUGR groups. Thus, although maternal age was matched, varying oxidative capacities of hUMSC might not have been taken into account. For instance, it has been shown that placentas obtained via caesarean section had increased inflammatory markers and oxidative stress than placentas obtained from spontaneous deliveries [76]. Although validated by repeated experiments, results from our *in vitro* experiments were based on pooled hUMSCs, which limits the ability to correlate our findings to the obtained clinical parameters. As another potentially limiting factor, hydrogen peroxide was used at concentrations shown to be biologically active in similar in vitro settings. However, since oxidative stress during pregnancy has been shown to increase in both physiological and pathological conditions [61], further experiments involving higher concentrations H_2_O_2_ remain to be performed. In this respect, however, it is known that H_2_O_2_ concentration higher than 500 microM is not able to affect the viability of trophoblasts [61]. Our study is further limited by its in vitro setup using third trimester hUSMCs in cross-talk with a first trimester trophoblast cell line. The influence of temporal origin of these cells might have differentially affected their direct and indirect response to the presented stimuli. Thus, further experiments with other trophoblast cell types and cell lines of different gestational age are needed to clarify such effects.
